# Benchmarking nurse outcomes in Australian Magnet® hospitals: cross-sectional survey

**DOI:** 10.1186/s12912-019-0383-6

**Published:** 2019-12-03

**Authors:** L. Stone, M. Arneil, L. Coventry, V. Casey, S. Moss, A. Cavadino, B. Laing, AL McCarthy

**Affiliations:** 1 Princess Alexandra Hospital, Woolloongabba, Queensland Australia; 2 University of Queenland, Brisbane, Queensland Australia; 3 Sir Charles Gairdner Hospital, Nedlands, Western Australia; 4 Edith Cowan University, Joondalup, Western Australia; 5 University of Queensland, Brisbane, Queensland Australia; 6 University of Auckland, Auckland, New Zealand; 7 Mater Health Services, South Brisbane, Queensland Australia

**Keywords:** Magnet®, Nursing outcomes, Job satisfaction, Burnout, Practice environment

## Abstract

**Background:**

Positive reports of nursing-related outcomes such as quality nursing care, nursing engagement with work and good practice environment are crucial in attaining and maintaining Magnet® designation. The majority of Magnet®-designated organisations (*N* = 482) are in the USA, with their aggregate nursing outcomes widely published as benchmark data. Australian Magnet® outcomes have not been aggregated or published to date.

**Methods:**

The aims are to benchmark educational preparation, occupational burnout, job satisfaction, intention to leave and working environment of nurses in Australian Magnet®-designated facilities and to determine the reliability of the Practice Environment Scale-Australia.

The design is a cross-sectional multisite survey set in all three Australian Magnet®-designated organisations.

The demographics included age, gender, level of education, years in practice, level of seniority and position title. Two items measured job satisfaction and intent to stay in current employment. The Maslach Burnout Inventory explored the three domains of nursing engagement: depersonalisation, personal achievement and emotional exhaustion. The Australian version of the Practice Environment Scale interrogated participants’ perceptions of their work environments.

**Results:**

2004 nurses participated (response rate 45.9%). Respondents’ mean age was 39.2 years (range 20–72). They were predominantly female and had worked in their current facility for more than 5 years. Eighty five percent had a minimum of a Bachelor’s degree. Eighty-six percent of respondents were satisfied or very satisfied with their current position. Eighty eight percent had no intention of leaving their current employer within the next 12 months. Participants rated their hospitals highly in all domains of the practice environment. Respondents reported less burnout in the personal accomplishment and depersonalisation domains than in the emotional exhaustion domain, in which they reported average levels of burnout. The internal consistency of the Practice Environment Scale-Australia was confirmed in this sample (Cronbach α’s 0.87–0.9 for subscales and 0.89 for composite score).

**Conclusion:**

In this paper, we present nursing outcome data from all Australian Magnet® hospitals for the first time. This provides a benchmark that facilitates comparison with nursing outcomes published by Australian non-Magnet® hospitals and with international Magnet® organisations.

## Background

Magnet® designation is conferred by the American Nurses’ Credentialing Centre (ANCC). Amongst other things, Magnet® designation indicates that a health service satisfies the ANCC’s criteria for nursing outcomes. Three important nursing outcomes are the capacity to attract and retain nurses who practise to the highest standards (which includes appropriate educational preparation), a high level of nursing engagement, and nurses’ perceptions of a good practice environment [1, 2]. Positive reports of these outcomes in American Magnet® hospitals are well documented [3]. In this paper, we present nursing outcome data from all Australian Magnet® hospitals for the first time.

While the critical mass of 482 [1] Magnet®-designated facilities is in the United States of America (US), Magnet® recognition is also sought internationally. Eight non-US hospitals currently hold Magnet® designation, three of which are Australian. The Australian hospitals comprise two government-funded ‘public’ facilities in Brisbane and Perth, and one ‘private’, not-for-profit hospital in Sydney.

Decades of work by the ANCC and affiliated researchers has led to considerable standardisation in how nursing outcomes are defined and assessed for designation and research purposes [3, 4]. Most research in this field assesses nurses’ educational preparation for practice, their job satisfaction and intention to remain in current employment, levels of burnout and perceptions of the quality of the nursing practice environment. This standardisation has facilitated comparison and bench-marking of these nursing outcomes across different US Magnet® settings [3, 4].

### The nursing practice environment

The nursing practice environment in Magnet® studies is usually defined as the organisational characteristics of a work setting that facilitate or impede professional nursing practice [5]. A good practice environment is distinguished by productive relationships between nurses, doctors, allied health and ancillary staff; meaningful nursing involvement in hospital affairs and devolved decision-making; hospital management that strives to continually improve the quality of patient care and responds to the concerns of nurses involved in that care; and investment in nursing professional development [6].

In Magnet® settings these factors are usually assessed with the Practice Environment Scale of the Nursing Work Index (PES-NWI). The US version of the PES-NWI is considered a valid and reliable instrument, with internal consistency coefficients originally reported for its five subscales as ranging from *α* = 0.71 (i.e., acceptable) to 0.85 (i.e., good) [5, 6] in the US. A recent study in Japan (*N* = 1219 PES respondents) indicates international reliability (Cronbach’s *α* ranging from 0.78 to 0.86 for subscales and 0.79 for the composite score) [7]. The 30-item Australian version (the PES-AUS) has one less item than the PES used in the US and elsewhere. Nursing diagnosis, which is part of the subscale ‘Nursing foundations for quality care’, is not included in the PES-AUS as nursing diagnoses are rarely, if ever, used in the Australian setting [8]. While the reliability coefficients of the PES-AUS were not reported by the modifiers of the instrument [8], the reliability of the original 31-item PES in Australia was reported in a study of 1192 nurses in the state of Queensland (composite score Cronbach’s *α* = 0.948; domain *α* range 0.705 to 0.892) [9]. This is problematic, because the Queensland study included the ‘nursing diagnosis’ item that is not used in any Magnet® facility in Australia. Hence the reliability of the PES-AUS commonly used by Australian Magnet® hospitals is not known.

### Nursing engagement: job satisfaction, turnover and burnout

Magnet® designation also indicates that nursing staff are engaged with their work. Engagement has three aspects. First engaged nurses’ express satisfaction with what they do. According to the ANCC [10], the high satisfaction of Magnet® nurses informs the second factor: engaged nurses intend to keep working at their facility. The combined effect of job satisfaction and intention to continue working with an organisation is low nursing turnover and high nursing retention.

A third factor that mediates job satisfaction and nursing retention is level of nursing burnout [2]. Occupational burnout is defined as a prolonged response to chronic work-related stressors [11]. Its three hallmarks are emotional exhaustion (feelings of being emotionally overextended and fatigued by one’s work); depersonalisation (an unfeeling and impersonal response towards the recipients of one’s care) and reduced personal accomplishment (the sense of competence and successful achievement) in individuals who work with other people [11]. In Magnet® studies, burnout is usually measured with the emotional exhaustion subscale of the Malachi Burnout Inventory-Human Service Survey (MBIHSS) [12]. The validity and reliability of the MBIHSS is internationally recognised [11].

The use of similar methods to define and assess critical Magnet® nurse outcomes has enabled pooling and benchmarking of Magnet® data in the US [3]. For example, the most recent aggregate report (published in 2011) indicated that compared to nurses employed in non-Magnet® facilities (*n* = 21,714), Magnet®-employed nurses (*n* = 4562) reported superior practice environments (*p* < 0.001), were more highly educated (*p* < 0.001), expressed less dissatisfaction with their employment (*p* < 0.05) and reported less emotional exhaustion (*p* < 0.05) [3]. It is timely to replicate US work on nursing outcomes. This would provide a useful international comparison with previously-published US Magnet® data, as well as a benchmark for any other Australian facilities considering the Magnet® journey.

## Aim of the study

The primary aim of this study was to provide a benchmark for educational preparation for practice, occupational burnout, job satisfaction, intention to leave and the hospital working environment in Magnet®-designated facilities in Australia. The secondary aim was to determine the reliability of the Practice Environment Scale-Australia (PES-AUS).

## Research design

This was a cross-sectional study undertaken in all three Magnet®-designated hospitals in Australia.

### Sample

All full-time or part-time registered nurses employed in the three Australian Magnet®-designated hospitals were eligible to participate. Magnet® designation is predicated on the outcomes of registered nurses involved in patient care and who have the security of longer-term employment. Therefore, nurses on casual contracts, Directors of Nursing and non-registered nurses (e.g., enrolled nurses, assistant nurses, and licensed vocational nurses) were excluded. There were 4368 registered nurses meeting these criteria when the study was undertaken. While we aimed to maximise response rates, this was an exploratory study and as such, sample size calculations were not indicated.

### Measures

#### Demographics

Demographics included age, gender, grade (type and seniority) of position held, highest nursing qualification obtained and years of employment in the current facility.

#### Nursing practice environment

The PES-AUS consists of 30 items that assess five domains. Each item asks participants to rate whether certain organisational characteristics are present using a 4-point Likert scale (1 = strongly disagree, 2 = somewhat disagree, 3 = somewhat agree, 4 = strongly agree). The ‘Nurse participation in hospital affairs’ subscale of nine items interrogates perceptions of nurses’ involvement in policy decisions, the access and visibility of senior nurses and career opportunities in the organisation. The nine items in the ‘Nursing foundations for quality of care’ subscale examines participants’ opportunities for continuing education and whether the organisation’s nursing standards are based on a defined model of care. The ‘Nursing unit manager ability, leadership and support of nurses’ subscale (five items) explores the degree to which senior nurses provide good leadership and a supportive work environment and recognise the achievements of their nurses. The fourth subscale (‘staffing resources adequacy’) elicits views of nurse-patient ratios, and time allocation for patient care and peer communication. The fifth subscale (‘collegial nurse-doctor relations’) seeks participants’ perceptions of the quality of nursing-medical teamwork in the organisation. Subscale scores are calculated by averaging individual responses to each item, while the overall score is calculated by averaging the five subscales.

#### Occupational burnout

Occupational burnout was measured with the 22 item MBIHSS. Respondents indicated on a 7-point Likert scale (0 = never, 1 = a few times a year, 2 = once a month or less, 3 = a few times a month, 4 = once a week, 5 = a few times a week, 6 = every day) the frequency with which they experienced certain feelings. The subscales of emotional exhaustion (nine items), depersonalisation (five items) and personal accomplishment (eight items) are not combined to report a composite score; rather, burnout is conceptualised as a continuous variable ranging from low to moderate degrees of the reported feeling. A high degree of burnout is reflected in higher scores on the emotional exhaustion and depersonalisation subscales, and low scores on the personal accomplishment scale [12]. An average degree of burnout is mirrored in average scores in all three subscales, while a low degree of burnout is reflected in low scores for the emotional exhaustion and depersonalisation subscales and high scores on the personal accomplishment subscale [11].

#### Job satisfaction and intention to leave

Following common practice in Magnet® studies [2], and to facilitate potential later pooling of data, job satisfaction was measured with one item. This asked participants to indicate how satisfied they were with their job on a 4-point Likert scale (very dissatisfied, somewhat dissatisfied, somewhat satisfied and very satisfied). Similarly, the final item (‘do you plan to leave your current employer in the next 12 months) offered two choices: ‘Yes, within the next year’ and ‘No plans within the next year’.

### Procedure

Prior to undertaking the study, the Executive Directors of Nursing (EDNS) of the three hospitals agreed that their staff could be approached to participate in the study. Human research ethics approval was obtained from each study site and the project team’s university. The study leads and project officer regularly discussed study progress with the Magnet® managers and research project staff at each site, to ensure consistency of study procedures and governance.

The Magnet® manager or research staff at each of the three sites sent an email to all eligible registered nurses through their human resource management system. The email contained a link to the online survey that invited eligible nurses to participate, described the purpose of the research and the requirements involved in completing the survey. Survey administration, which was undertaken electronically through Survey Monkey Inc., was staggered between July and November 2016, with each site undertaking data collection for 6 weeks. Due to different human resource management systems, each site managed their own recruitment and administered the survey through separate Survey Monkey Inc. platforms. Completion of the survey implied consent. To enable follow up of participants, all potential participants were assigned a unique identifier code, the coding key for which was kept by the site-specific project officer in a secure location.

A reasonable window of opportunity enabled the participants to complete the survey, after which reminder emails were sent at regular intervals to improve response rates [13]. To maximise response rates and encourage a sense of competition, each site collated their unit-specific aggregate response rates each week and filtered them down to staff through hospital-appropriate channels for encouragement. All respondents were entered into a ‘lucky draw’, with 5 respondents from each hospital (*N* = 15) winning two movie vouchers.

### Data analysis

Data from each site were cleaned and harmonised by two project staff. The disparate nursing position titles from each State were harmonised into the four Queensland bands of Grade 5 (base grade registered nurse), Grade 6 (nurse recognised for more advanced specialty skills), Grade 7 (an advanced practice nurse such as a clinical nurse consultant, nurse researcher, nurse educator or nurse unit manager) and Grade 8 (nurse practitioner).

Analysis was performed using Stata v.15. For descriptive statistics, categorical variables are presented as counts and percentages and continuous variables are presented using means and standard deviation. Logistic regression examined potential associations of age (continuous), gender and nursing classification with job dissatisfaction (very/somewhat dissatisfied versus somewhat/very satisfied), intent to leave in the next 12 months and high levels of burnout. High burnout was defined according to MBIHSS domains as high (vs. low/moderate) emotional exhaustion or depersonalisation, and low (vs. moderate/high) personal achievement. Models were adjusted for age, gender, nursing classification and each site in order to take account of hospital-level differences. The internal validity of PES-AUS scores was assessed using Cronbach’s *α*, where values > 0.7 are taken as acceptable indicators of scale reliability. Logistic regression examined associations between the PES-AUS scores and job dissatisfaction, intention to leave and high levels of nurse burnout, with models adjusted for hospital site, age, gender and nursing grade. Complete case analysis was used throughout, such that the numbers included differ in each analysis. Results are presented as odds ratios (ORs) and 95% confidence intervals (95% CIs) in table or figure format.

## Results

Most questions were optional; hence the data represent those nurses who chose to respond to a particular question. A total of 2004 nurses meeting the inclusion criteria responded to the survey request, equating to a response rate of 45.9%. Table 1, which profiles the demographic characteristics of respondents, indicates a mean age of 39.2 years (range 20–72). The sample comprised a predominantly female workforce who had worked in their current facility for more than 5 years. Most (85.2%) had a minimum of a Bachelor's degree. The demographics of this sample were consistent with those of the national nursing workforce [14], except for age. The mean age of the Australian nurse was 44.5 years while the mean age of the Magnet sample was 39.2 years (range 20–72) [14].

**Table 1:** Demographics

	N	%	Mean (SD)
Gender	2004		
Female	1724	86.03	
Male	203	10.13	
I’d prefer not to say	77	3.84	
Age (range 20–72)	1885		39.17 (11.38)
Under 25	212	11.25	
25–34	547	29.02	
35–44	484	25.68	
45–54	439	23.29	
55–64	180	9.55	
65–74	23	1.22	
Didn’t respond	119	5.91	
Nursing grade^a^	2004		
Grade 5	1256	62.67	
Grade 6	433	21.61	
Grade 7	301	15.02	
Grade 8	14	0.70	
Highest nursing qualification	1965		
Industry qualification (hospital-acquired)	150	7.63	
Vocational education sector qualification (e.g. technical college)	140	7.12	
Undergraduate degree	1040	52.93	
Postgraduate certificate/diploma (university level)	473	24.07	
Masters and above	162	8.24	
Didn’t respond	39	1.95	
Years in current facility (range 0–43)	1953		6.74 (6.61)
Less than 2 years	464	23.76	
2 – under 5 years	444	22.73	
5 – under 10 years	567	29.03	
10 – under 20 years	352	18.02	
20 years and over	126	6.45	
Didn’t respond	51	2.54	

^a^Nursing levels and titles differ between the 7 Australian jurisdictions. Grades were standardised to equate to the Queensland model, where increasing grade denotes increasing seniority and/or specialisation. Grade 5 = base grade registered nurse, Grade ^14^6 is usually more advanced specialty skills, Grade 7 is an advanced practice nurse e.g. nurse unit manager, clinical nurse consultant, senior clinical specialist, nurse educator or nurse researcher. Grade 8 is a nurse practitioner

### Job satisfaction and intent to leave

Most respondents (*n* = 1621, 80.9%) to the item concerning job satisfaction were satisfied with their current position (4.8% very dissatisfied, 9.4% somewhat dissatisfied, 48.4% satisfied and 37.4% very satisfied). However, 383 (19.1%) nurses did not respond to the question around job dissatisfaction, and these nurses were 3 years older on average (mean difference = 2.9 years, 95% CI 1.6 to 4.2, t-test *p*-value< 0.001). In addition, a significantly higher proportion of female (19.9% vs. 13.8% vs of male nurses, *p* = 0.04) and grade 7–8 (33.3% vs. 16.5% of grade 5–6 nurses, *p* < 0.001) nurses did not respond to questions around job satisfaction. Of the 1983 participants (98.9%) who responded to the item asking whether they planned to leave their current employer in the next 12 months, 11.8% responded ‘yes’ compared to 88.2% responded ‘no’.

### Nursing burnout

**Fig. 1:**
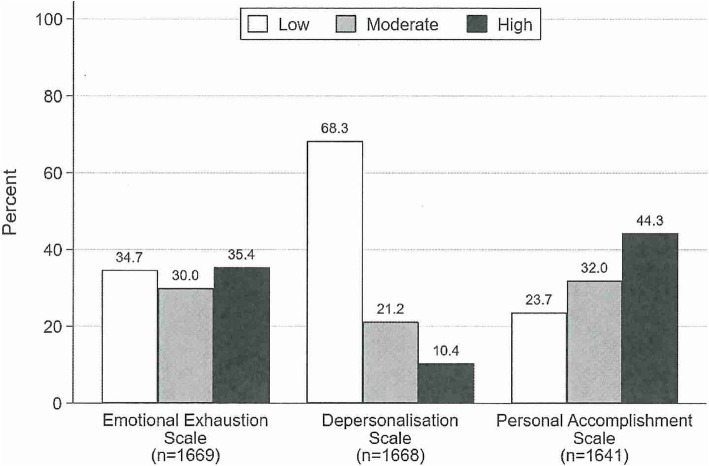
Categorisation of the Maslach Burnout Inventory Human Services Survey (MBIHSS)

As all questions in this survey were optional, the total number of respondents for each of the MBIHSS domains was not the same. At least one of the three MBIHSS subscales was available for 260 nurses (13.0%), with 1525 nurses (76.1%) answering all MBIHSS questions. There were no age or gender differences in those not responding to questions relating to MBIHSS. Figure 1 indicates respondents reported approximately equal perceptions of low (34.7%), moderate (30.0%) and high (35.4%) levels of emotional exhaustion; the majority reported low (68.3%) levels of depersonalisation, and the majority reported high (44.3%) levels of personal accomplishment.

### Association of demographic factors with job dissatisfaction, intent to stay and nursing burnout

Table 2 presents results from the logistic regression models that explored the potential associations between respondents’ age, gender and grade with job dissatisfaction, intent to leave and levels of job-related burnout. For each 10-year increase in age, respondents were 16% more likely to report job dissatisfaction, but were 21% less likely to express an intention to leave within 12 months. A 10-year increase in age was also associated with a 23% decrease in the odds of having reported high levels of emotional exhaustion and a 32% decrease in the odds of reporting high levels of depersonalisation. There was no association between respondents’ age and their reported levels of personal accomplishment. Gender was not associated with job dissatisfaction or intention to leave, although male nurses were 67% more likely to report high levels of emotional exhaustion and had around twice the odds of high levels of depersonalisation and low levels of personal achievement. Grade 7–8 nurses were less likely to express an intention to leave and reported lower levels of burnout (for all three domains) compared to Grade 5–6 nurses. However, after additionally adjusting for age and gender, associations between nursing grade and intention to leave, emotional exhaustion and depersonalisation were not statistically significant. Grade 7–8 nurses were 48% less likely to report low levels of personal accomplishment than lower grade nurses.

**Table 2:** Association of demographic factors with job dissatisfaction, intent to stay and job-related burnout

Variable	Comparison	Univariate models^a^		Multivariate models^a^	
		OR (95% CI)	*P*-value	OR (95% CI)	*P*-value
Job dissatisfaction					
Age	per 10 year increase	1.14 (1.00–1.29)	0.05	1.16 (1.01–1.33)	0.03
Gender	Male vs female	1.03 (0.65–1.63)	0.91	0.95 (0.59–1.55)	0.85
Nursing grade	Grade 7–8 vs 5–6	0.73 (0.46–1.16)	0.19	0.68 (0.41–1.13)	0.14
Intent to Leave					
Age	per 10 year increase	0.76 (0.67–0.87)	< 0.001	0.79 (0.68–0.90)	0.001
Gender	Male vs female	1.14 (0.73–2.79)	0.56	1.02 (0.63–1.64)	0.94
Nursing grade	Grade 7–8 vs 5–6	0.58 (0.37–0.91)	0.02	0.66 (0.40–1.09)	0.10
High emotional exhaustion					
Age	per 10 year increase	0.75 (0.68–0.82)	< 0.001	0.77 (0.69–0.85)	< 0.001
Gender	Male vs female	1.66 (1.20–2.29)	0.002	1.67 (1.20–2.33)	0.002
Nursing grade	Grade 7–8 vs 5–6	0.66 (0.49–0.88)	0.005	0.83 (0.60–1.16)	0.28
High depersonalisation					
Age	per 10 year increase	0.64 (0.55–0.75)	< 0.001	0.68 (0.68–0.80)	< 0.001
Gender	Male vs female	2.14 (1.38–3.31)	0.001	2.07 (1.31–3.23)	0.002
Nursing grade	Grade 7–8 vs 5–6	0.42 (0.23–0.74)	0.003	0.56 (0.29–1.08)	0.08
Low personal accomplishment					
Age	per 10 year increase	0.98 (0.88–1.09)	0.71	1.04 (0.94–1.16)	0.45
Gender	Male vs female	1.99 (1.40–2.81)	< 0.001	1.97 (1.36–2.78)	< 0.001
Nursing grade	Grade 7–8 vs 5–6	0.55 (0.38–0.79)	0.001	0.52 (0.35–0.78)	0.001

^a^Individual models are separate logistic regression models for each outcome and each IV, adjusted for site; multivariate models include age, gender, nursing classification and site in a logistic regression for each outcome

### The nursing practice environment

As indicated in Table 3, respondents rated their hospitals highly in all domains of the practice environment and overall. The composite PES-AUS scale was calculated for the 1761 nurses (91.8%) who responded to all PES-AUS-related questions. The internal consistency of the PES-AUS was confirmed in this sample, with high Cronbach *α’s,* in the range 0.87–0.9 for all subscales and 0.89 for the composite score.

**Table 3:** Practice Environment Scale - Australia (PES-AUS) scores and scale reliability

Domain	Number of sub-items	N	Mean	SD	Range	Cronbach’s *α*
Nurse participation in hospital affairs	9	1726	2.95	0.59	2.91–3.22	0.90
Nursing foundations for quality of care	9	1693	3.23	0.48	3.18–3.41	0.87
Nurse manager ability, leader ship and support of nurses	5	1772	3.14	0.65	3.07–3.29	0.87
Staffing and resource adequacy	4	1754	2.92	0.68	2.80–2.99	0.86
Collegial nurse-physician relationships	3	1799	3.19	0.59	3.15–3.26	0.86
Composite	5	1761	3.09	0.5	3.02–3.24	0.89

**Fig. 2:**
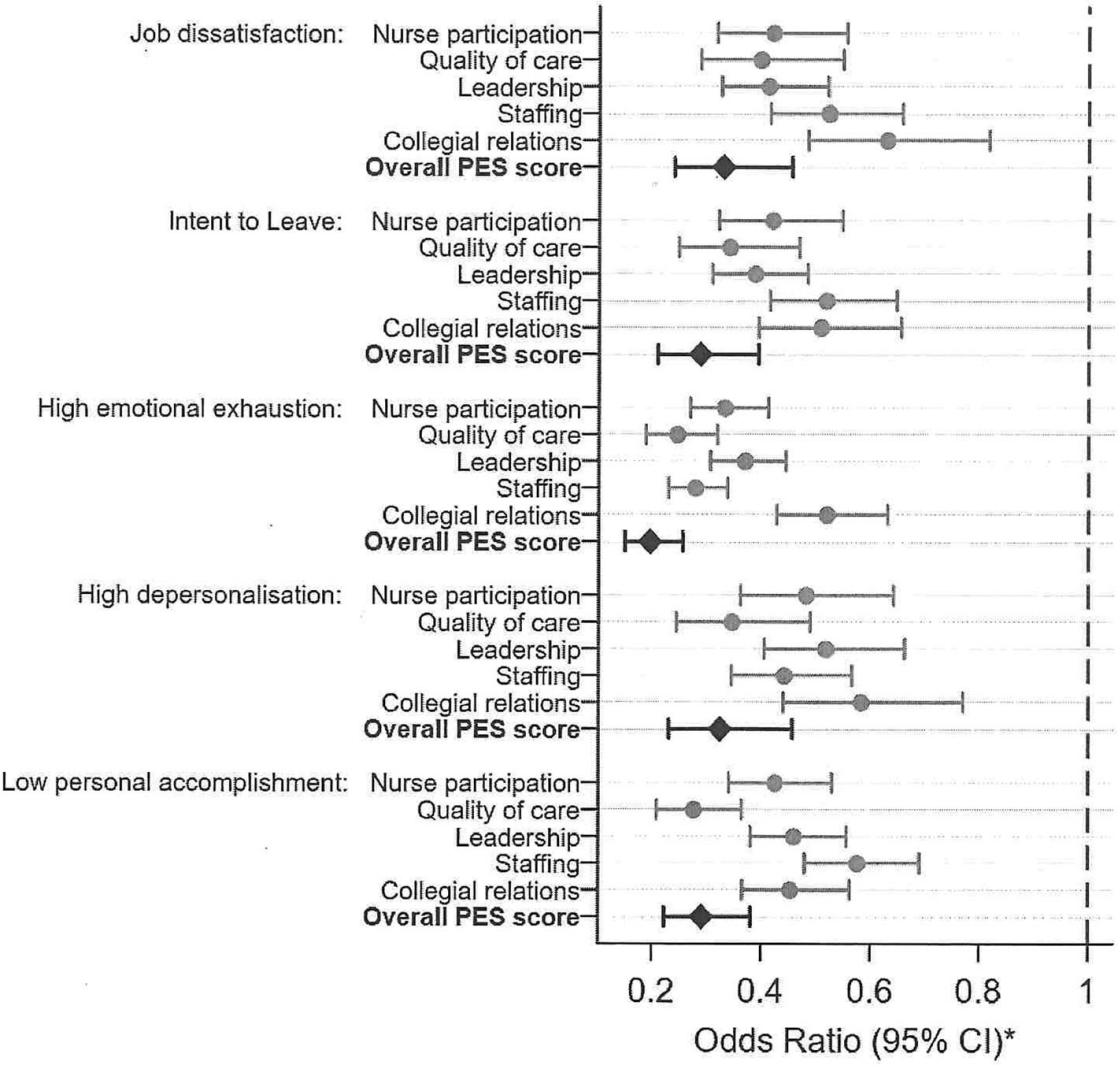
Association of PES-AUS scores. * Logistic regression adjusted for hospital site, age, gender and nursing grade. ** An odds ratio of 1 represents no effect, indicated here with a blue dashed line. *** Scores were with: job dissatisfaction, intent to leave, high emotional exhaustion, high depersonalisation and low personal accomplishment

Figure 2 indicates that higher scores on the five PES-AUS subscales and the composite scale were consistently and strongly associated with lower levels of job dissatisfaction, less intention to leave and less job-related burnout (*p* < 0.001 for all models). Odds ratios for these associations ranged from 0.2 to 0.6, representing 40–80% lower odds of job dissatisfaction, intention to leave and burnout for each one-unit increase in the mean for the PES scale included in that model.

## Discussion

Magnet® hospital recognition is often (but not always) associated in the literature with lower nurse turnover, less nurse burnout, greater job satisfaction, more advanced preparation for practice and a better working environment than non-Magnet® facilities [2, 3]. The results of this study provide a benchmark for these outcomes in the Australian Magnet® setting. The results also confirm that the PES-AUS measures those aspects of the practice setting that it purports to measure.

### Educational preparation for practice

Magnet® status indicates that the organisation encourages and enables its nurses to undertake education and development through every stage of their career. To meet the Magnet® standards, this means that nurses are educationally-prepared to practise. The standards stipulate that 100% of unit-based nurse managers and the hospital-wide nursing executive must have a minimum of a Bachelor’s degree; that the Executive Director of Nursing has a minimum of a Master’s degree; and that the credentialing rate of nurses working in advanced or specialty roles increases annually. The majority (85%) of respondents, including the large proportion of base-grade registered nurses, held a minimum of a Bachelor’s degree. This result was anticipated. Hospital-based education was phased out in Australia 30 years ago, with university education the only mechanism leading to registration since the early 1990s. Given the mean age of the Australian Magnet cohort (39.18 years) it would be expected that most participants would hold a minimum of a baccalaureate degree. Nearly a third (32.3%) of respondents held a postgraduate qualification, but these data are more difficult to interpret. They cannot be compared to international Magnet® data, or to non-Magnet hospital data in Australia, for two reasons.

First, the educational preparation of its registrants is not recorded by the Australian national nurse registering body; hence it is impossible to estimate how many practising nurses in Australia possess post-basic qualifications for comparison. Second, even when it is attainable, it is difficult to compare Australian post-basic data with data from international Magnet® hospitals, the majority of which are in the US. The Australian Qualifications Framework differs markedly from the US post-registration structure, which inhibits the ability to compare performance in postgraduate qualifications. For example, specialty ‘credentialing’ is largely an American concept. It is not a term widely-used in Australia, it is not a requirement for career advancement nor is it regulated by a professional nursing organisation. The exceptions are nurse practitioners and mental health nurses, whose post-registration education is regulated and who are ‘endorsed’ rather than credentialed. In essence, it is Australian universities (not professional or nursing regulatory bodies) that confer specialty qualifications in areas like intensive care and oncology nursing and unlike the US, these qualifications do not require annual updating. Comparing postgraduate qualifications and credentials is therefore not possible.

The Australian approach to specialty and advanced practice is clearly not aligned with US-developed Magnet® expectations and can be difficult for US Magnet® representatives to evaluate during the designation process. The data presented here are therefore a benchmark for future Magnet® studies in Australia but at present are not useful for comparative purposes with Magnet® nursing outcomes in other countries.

### Job satisfaction and intention to stay

An original and enduring focus of the Magnet® program is nurses’ job satisfaction, with concomitant emphasis on the recruitment of high-quality staff and reduction in nursing turnover. Magnet® designation is often associated with superior outcomes in recruitment, retention and job satisfaction in the US compared to non-Magnet® hospitals. Whilst achieving and retaining Magnet® designation is a costly exercise, high nursing turnover is similarly expensive. It is argued that, particularly in larger facilities, the lessening of costs associated with decreased turnover of high quality staff more than compensates for the expense of pursuing and maintaining Magnet® designation [2].

While we detected a trend for older nurses to express more job dissatisfaction than younger nurses, in the Australian Magnet® hospitals studied here most nurses expressed satisfaction with their current employment and did not indicate retention was problematic. Due to the non-interventional nature of this study, however, it is not clear whether this is attributable to Magnet® designation or is an artefact of Australian working conditions. For example, the two most recent large surveys of nursing job satisfaction in Australia indicate a general trend towards job satisfaction and intention to stay in Australian nurses. One study of 2000 Australian registered nurses reported that 81% were satisfied in their jobs [15]. Another sample of 562 nurses indicated that 96% were moderately or highly satisfied with their current employment [16]. It could be that the high level of job satisfaction of Australian nurses generally is a result of their working conditions rather than their employers’ Magnet® status. Compared to US nurses (including those employed in Magnet® facilities) Australian nurses often have legislated nurse-patient ratios of 1:4 during day shifts in acute environments [17, 18]. Australian nurses working in government-funded facilities also have more favourable leave entitlements than US nurses, including 4 weeks annual leave from the date of employment, 5 weeks annual leave per year for those working shifts across 24 h from the date of employment, time-and-a-half paid for ‘after hours’ and weekend shifts, ‘double time’ for public holidays, on-call allowances, fatigue leave, accrued days off and time-off-in-lieu, up to 5 days mandated professional development leave, 10 days paid sick leave annually, and 12 weeks paid long service leave after 10 years of employment [19]. There is also significantly more generous parental leave. While this varies from state to state, it is not unusual to be granted 12 months parental leave, with up to 18 weeks of this fully paid [19].

### The practice environment

Magnet® hospitals are associated with a better quality of nursing care than matched controls [20], a finding mediated in one comparative study of 56 Magnet® and 495 non-Magnet® hospitals in the US by the “superiority” of the Magnet® hospitals’ practice environment [20]. The results of this study indicate that Australian nurses employed in Magnet® facilities report an even better practice environment than their Magnet® counterparts in other countries. In their integrative review of PES-NWI data from studies conducted in 28 other countries [4], Swiger et al. noted that Magnet® organisations scored higher for the practice environment than both non-Magnet® and “aspiring Magnet®” facilities. Yet the Australian Magnet results are higher still in all domains of the PES-AUS and in the composite score than those collated by Swiger et al. [4] They are similarly higher than the results reported in the only Australian study investigating this issue with the PES in non-Magnet® settings [9].

While the non-interventional nature of the present study precludes firm attribution of these results to Magnet® designation, it is tempting to argue that the reported excellence of the practice environments explored here is likely a result of the longevity of Magnet® and its value to the leadership and staff of these three organisations. One of the hospitals in this review was in the final phase of its fourth Magnet® accreditation and has 17 years of Magnet® experience; one was preparing for its third designation, and the other has been on the Magnet journey since 2009. Such experience with the designation process could indicate that, in line with Magnet® standards, the Magnet® principles of shared governance are firmly embedded in these organisations, that their governance structures are relatively flat and consultative, and that educational opportunities and nursing autonomy are valued and promoted [21].

### Burnout

There is considerable global evidence from large studies that nurses experience high levels of burnout [3, 22–25]. There is also a significant body of evidence associating burnout with poorer quality health care, high job turnover, low morale [11], and poor engagement with nursing work [23]. Hence the emphasis in Magnet® studies on understanding and mitigating burnout. Aggregate data from the US indicate that Magnet®-employed nurses are 13% less likely to report emotional exhaustion as measured by the MBIHSS [3]. The present study, wherein respondents reported relatively low proportions of burnout in the depersonalisation and personal accomplishment domains, and approximately equal proportions of low, moderate and high emotional exhaustion, provides the benchmark for further Magnet® studies in Australia.

In contrast to most Magnet® studies, which only administer the emotional exhaustion sub-scale, in this study the full MBIHSS was administered. Recent research by the developer of the MBIHSS [26] indicates that this practice of partial instrument administration should be reconsidered. Burnout is not the equivalent of emotional exhaustion but a complex interplay of emotional exhaustion with other work-related issues [26]: the individual experience of work-related stress occurs within the social context [11, 27]. This means that burnout is multidimensional. Different and distinct profiles emerge when all MBIHSS scales are administered [26], all of which correlate differently with organisational variables and require different organisational interventions when identified [27, 28], such as strategies to manage workplace demands or to develop resilience [26]. The instrument developers now caution against the use of emotional exhaustion alone as a proxy for burnout and advise that all three scales of the instrument are administered [26].

## Limitations

This was a cross-sectional non-interventional survey study offering a snapshot of Magnet nurses at one point in time. Given that we did not manipulate participants’ working environments or study their perceptions over time, we cannot determine cause and effect relationships between variables. A further limitation is the self-reported nature of the data: self-report does not always result in accurate reporting. For example, the participants might have been motivated to provide responses they judged as more socially acceptable in the Magnet context. There was considerable item non-response for some questions, and so we cannot rule out potential biases; for example, in the observed levels of reported job satisfaction and nursing burnout. Biases might occur if the reason for a nurse not answering a particular question was related to how that question would have been answered. For example, we observed that nurses reporting job dissatisfaction were older on average and also that those who did not answer the question regarding job satisfaction were older on average. It is therefore possible that the levels of job dissatisfaction reported here were underestimated. Finally, a response rate of 40%, while reasonable, might have resulted in response bias. We do not know the characteristics of the 60% of non-responders, whether they differed in any way from participants, and whether their responses would have taken the results in another direction.

## Conclusion

In this study, we determined the profile of Australian nurses practising in Magnet®-designated organisations, the characteristics of their practice environments, and their engagement with their work for the first time. While no claims for causality are made, the results indicate that Magnet®-employed nurses in Australia report job satisfaction and intend to continue their employment. They also experience a better working environment than their international colleagues, average levels of emotional exhaustion, low levels of depersonalisation and good levels of personal accomplishment. The data from this study provide a benchmark for future Magnet® studies undertaken in Australia and internationally.

## Data Availability

The datasets used and analysed during the current study are available from the corresponding author on reasonable request.

## Abbreviations

ANCC: American Nurses’ Credentialing Centre
EDNS: Executive Directors of Nursing
MBIHSS: Maslach Burnout Inventory-Human Service Survey
PES-AUS: Practice Environment Scale-Australia
PES-NWI: Practice Environment Scale of the Nursing Work Index
STROBE: Strengthening the Reporting of Observational Studies in Epidemiology

## References

[1] American Nurses Credentialing Center (2018). Magnet Recognition Program. ANCC 2019.

[2] Kutney-Lee A, Stimpfel AW, Sloane DM, Cimiotti JP, Quinn LW, Aiken LH (2015). Changes in patient and nurse outcomes associated with magnet hospital recognition. Med Care.

[3] Kelly LA, McHugh MD, Aiken LH (2012). Nurse outcomes in magnet(R) and non-magnet hospitals. J Nurs Adm.

[4] Swiger PA, Patrician PA, Miltner RSS, Raju D, Breckenridge-Sproat S, Loan LA (2017). The practice environment scale of the nursing work index: an updated review and recommendations for use. Int J Nurs Stud.

[5] Kutney-Lee A, Lake ET, Aiken LH (2009). Development of the hospital nurse surveillance capacity profile. Res Nurs Health.

[6] Aiken LH, Cimiotti JP, Sloane DM, Smith HL, Flynn L, Neff DF (2011). Effects of nurse staffing and nurse education on patient deaths in hospitals with different nurse work environments. Med Care.

[7] Ogata Y, Sasaki M, Yumoto Y, Yonekura Y, Nagano M, Kanda K (2018). Reliability and validity of the practice environment scale of the nursing work index for Japanese hospital nurses. Nursing open.

[8] Walker K, Middleton S, Rolley J, Duff J (2010). Nurses report a healthy culture: results of the practice environment scale (Australia) in an Australian hospital seeking magnet recognition. Int J Nurs Pract.

[9] Parker D, Tuckett A, Eley R, Hegney D (2010). Construct validity and reliability of the practice environment scale of the nursing work index for Queensland nurses. Int J Nurs Pract.

[10] ANCC (2019). Why Become Magnet.

[11] Maslach C, Leiter MP (2016). Understanding the burnout experience: recent research and its implications for psychiatry. World Psychiatry.

[12] Maslach C, Jackson SE, Leiter MP. Maslach Burnout Inventory: Manual and Non-Reproducible Instrument and Scoring Guides. Menlo Park: Mind Garden; 2010.

[13] Dillman DA, Smyth JD, Christian LM. Internet, Phone, Mail, and Mixed-Mode Surveys: The Tailored Design Method. Hoboken: Wiley; 2014.

[14] Australian Institute of Health and Welfare (2015). Nursing and Midwifery Workforce 2015.

[15] Reid C, Hurst C, Anderson D (2013). Examination of socio-demographics and job satisfaction in Australian registered nurses. Collegian.

[16] Skinner V, Madison J, Humphries JH (2012). Job Satisfaction of Australian Nurses and Midwives: A Descriptive Research Study. Aust J Adv Nurs.

[17] Queensland Health (2019). Nurse-to-Patient Ratios.

[18] Victorian Government Australia (2015). Safe Patient Care (Nurse to Patient and Midwife to Patient Ratios) Act 2015 no. 51.

[19] Queensland Health 2019 (2019). Leave Entitlements.

[20] Stimpfel AW, Rosen JE, McHugh MD (2014). Understanding the role of the professional practice environment on quality of Care in Magnet(R) and non-magnet hospitals. J. Nurs Adm.

[21] America Nurses Credentialing Center 2019 (2019). Magnet Model.

[22] Kanai-Pak M, Aiken LH, Sloane DM, Poghosyan L (2008). Poor work environments and nurse inexperience are associated with burnout, job dissatisfaction and quality deficits in Japanese hospitals. J Clin Nurs.

[23] Moloney W, Boxall P, Parsons M, Cheung G (2018). Factors predicting registered nurses’ intentions to leave their organization and profession: a job demands-resources framework. J Adv Nurs.

[24] Nantsupawat A, Srisuphan W, Kunaviktikul W, Wichaikhum O, Aungsuroch Y, Aiken LH (2011). Impact of nurse work environment and staffing on hospital nurse and quality of Care in Thailand. J Nurs Scholarsh.

[25] Van Bogaert P, Kowalski C, Weeks SM, Clarke SP (2013). The relationship between nurse practice environment, nurse work characteristics, burnout and job outcome and quality of nursing care: a cross-sectional survey. Int J Nurs Stud.

[26] Leiter MP, Maslach C (2016). Latent burnout profiles: a new approach to understanding the burnout experience. Burn Res.

[27] Li B, Bruyneel L, Sermeus W, Van den Heede K, Matawie K, Aiken L, Lesaffre E (2013). Group-level impact of work environment dimensions on burnout experiences among nurses: a multivariate multilevel Probit model. Int J Nurs Stud.

[28] Casalicchio G, Lesaffre E, Küchenhoff H, Bruyneel L (2017). Nonlinear analysis to detect if excellent nursing work environments have highest well-being. J Nurs Scholarsh.

